# Presepsin as a predictor of septic shock in patients with urinary tract infection

**DOI:** 10.1186/s12894-021-00906-4

**Published:** 2021-10-12

**Authors:** Yoshitaka Sekine, Kazuhiko Kotani, Daisuke Oka, Hiroshi Nakayama, Yoshiyuki Miyazawa, Takahiro Syuto, Seiji Arai, Masashi Nomura, Hidekazu Koike, Hiroshi Matsui, Yasuhiro Shibata, Masami Murakami, Kazuhiro Suzuki

**Affiliations:** 1 Department of Urology, Gunma University Graduate School of Medicine, 3-39-22 Showa-Machi, Maebashi, 371-8511 Japan; 2 Division of Community and Family Medicine, Department of Clinical Laboratory Medicine, Jichi Medical University, Shimotsuke, Japan; 3 Department of Clinical Laboratory Medicine, Gunma University Graduate School of Medicine, Maebashi, Japan

**Keywords:** Bacterial infections, Biomarkers, C-reactive protein, Cytokines, Endotoxins, Flank pain, Presepsin, Urinary tract infection, Sepsis; procalcitonin

## Abstract

**Background:**

Recently, presepsin has been reported to be a useful biomarker for early diagnosis of sepsis and evaluation of prognosis in septic patients. However, few reports have evaluated its usefulness in patients with urinary tract infections (UTI). This study aimed to evaluate whether presepsin could be a valuable marker for detecting severe sepsis, and whether it could predict the therapeutic course in patients with UTI compared with markers already used: procalcitonin (PCT) and C-reactive protein (CRP).

**Methods:**

From April 2014 to December 2016, a total of 50 patients with urinary tract infections admitted to Gunma university hospital were enrolled in this study. Vital signs, presepsin, PCT, CRP, white blood cell (WBC) count, causative agents of urinary-tract infections, and other data were evaluated on the enrollment, third, and fifth days. The patients were divided into two groups: with (n = 11) or without (n = 39) septic shock on the enrollment day, and with (n = 7) or without (n = 43) sepsis on the fifth day, respectively. Presepsin was evaluated as a biomarker for systemic inflammatory response syndrome (SIRS) or septic shock.

**Results:**

Regarding the enrollment day, there was no significant difference of presepsin between the SIRS and non-SIRS groups (*p* = 0.276). The median value of presepsin (pg/mL) was significantly higher in the septic shock group (*p* < 0.001). Multivariate logistic regression analysis showed that presepsin (≥ 500 pg/ml) was an independent risk factor for septic shock (*p* = 0.007). ROC curve for diagnosing septic shock indicated an area under the curve (AUC) of 0.881 for presepsin (vs. 0.690, 0.583, and 0.527 for PCT, CRP and WBC, respectively). Regarding the 5th day after admission, the median presepsin value on the enrollment day was significantly higher in the SIRS groups than in the non-SIRS groups (*p* = 0.006). On the other hand, PCT (≥ 2 ng/ml) on the enrollment day was an independent risk factor for SIRS. ROC curve for diagnosing sepsis on the fifth day indicated an AUC of 0.837 for PCT (vs. 0.817, 0.811, and 0.802 for presepsin, CRP, and WBC, respectively).

**Conclusions:**

This study showed that presepsin may be a good marker for diagnosing septic shock based on admission data in patients with UTI.

**Supplementary Information:**

The online version contains supplementary material available at 10.1186/s12894-021-00906-4.

## Background

Urinary tract infections (UTI) are common, and sometimes progress to sepsis or septic shock, which can be lethal. Mortality rate due to severe sepsis and septic shock has been reported to be between 20 and 50% [1, 2], and 9–31% of all cases of severe sepsis and septic shock has been reported to arise from UTI [2]. Therefore, diagnosis and evaluation of severity of sepsis or septic shock are important at the beginning of treatment of UTI.

Clinically, C-reactive protein (CRP) and procalcitonin (PCT) are used as markers for disease severity in patients with UTI. However, both CRP and PCT have some limitations. The response time to bacterial infections is delayed (CRP, 6 h; PCT, 2–3 h), production triggers might not be living bacteria (CRP, cytokine; PCT, endotoxin and cytokine), and serum half-time is long (CRP, 4–6 h; PCT, 20–24 h) [3, 4]. Therefore, a novel biomarker for bacterial infection, which reflects clinical condition at the time of measurement, is required.

Presepsin is a 13KDa N-terminal fragment of soluble CD14 [5]. Granulated leukocytes phagocytose both bacteria and CD14 and expel presepsin into the blood after enzymatic digestion of bacteria within two hours [5, 6]. Recently, presepsin has been reported to have a high sensitivity in detecting sepsis and be a biomarker for early diagnosis of sepsis [7]. Moreover, elevated presepsin levels on day one might evaluate prognosis of patients with sepsis in intensive-care units [8, 9]. However, there are a few reports on the use of presepsin as a biomarker in patients with UTI.

This study aimed to evaluate whether presepsin could be a useful marker for detecting sepsis or severe sepsis, and whether it could predict therapeutic courses in patients with UTI compared with other markers, such as PCT or CRP.

## Methods

### Patients

We performed a prospective observational study. From April 2014 to December 2016, a total of 57 patients with UTI, who were admitted into Gunma university hospital, were enrolled in this study. Seven patients were excluded from this study due to data unavailability. The urologist diagnosed UTI based on urinary sediment (≥ 5 leucocytes/high power field) and symptoms (fever and/or micturition pain and/or flank pain). Data of patient age, sex, medical history, oral medicine, blood pressure, body temperature, heart rate, respiratory rate, urine and blood culture results, surgical procedure for UTI after admission, causative agents of UTI, presepsin, PCT, CRP, white blood cell (WBC), Aspartate transaminase (ALT), Alanine transaminase (AST), γ-glutamyl transpeptidase (γGPT), and Creatinine (Cr) were collected. Vital signs and blood test results were evaluated at enrollment, and on the 3rd and 5th day after admission. The written informed consent was obtained from all of the enrolled patients. This study was approved by the Institutional Review Board of Gunma University Hospital (No.1650).

### Assessment of systemic inflammatory response syndrome (SIRS) and septic shock

Diagnosis of SIRS and septic shock were established according to the criteria set by the American College of Chest Physicians/Society of Critical Care Medicine (ACCP/SCCM) [10].

### Outcomes

Three outcome variables: SIRS and septic shock on the enrollment day and SIRS on the 5th day after admission were evaluated by dividing the participants into two groups for each of these variables; with (n = 39) or without (n = 11) SIRS on the enrollment day, with (n = 11) or without (n = 39) septic shock on the enrollment day, and with (n = 7) or without (n = 43) SIRS on the 5th day after admission, respectively.

### Statistical analysis

Mann–Whitney U-test was used for continuous variables (age, CRP, presepsin, PCT, ALT, AST, γGPT, and Cr). We estimated independence using the chi-squared test or Fisher`s exact test for categorical variables (sex, placement of urinary catheter, urological cancer, urinary calculi, diabetes mellitus, and internal use of steroids). Independent predictors were evaluated using logistic regression analysis. The predictive accuracy of presepsin, PCT, CRP and WBC for septic shock or SIRS was evaluated by the area under the curve (AUC) of receiver operating characteristics (ROC) analysis. The Youden's index (sensitivity + specificity—1) was used to calculate optimal cutoff values for presepsin. *P* values  ≤ 0.05 were considered statistically significant. SPSS Statistics Ver. 25 (IBM Corp. IL, USA) was used for statistical analyses.

## Results

### Patients characteristic

Table 1 shows the clinical characteristics of the 50 patients. On the enrollment day, septic shock was detected in 22% (n = 11) and SIRS in 78% (n = 39) of the patients. The mean age was 66.5 years, and 33 patients (66%) were men and 17 (34%) were women. Urine and blood cultures were positive and revealed bacterial growth in in 90% (n = 45) and 48% (n = 24) of samples, respectively. Forty-eight% (n = 24), 30% (n = 15), 24% (n = 12), 18% (n = 9), 56% (n = 28), and 18% (n = 9) were with placement of urinary catheter, urological cancer, urinary calculi, diabetes mellitus, surgical procedure after admission, and internal use of steroids, respectively.

**Table 1:** Characteristics of Patients with or without septic shock on the enrollment day

Variable	Total	Septic shock group	Non-septic shock group	*p *value
	N = 50	N = 11	N = 39	
Age (years)	66.5 [60.3–75.5]]	64 [49–70]	68 [61–78]	0.055
White blood cell (/mm^3^)	13,600 [10,375–18,000]	13,600 [9900–25600]	13,600 [10400–16600]	0.788
C-reactive protein (mg/L)	10.45 [4.26–23.06]	11.47 [6.41–25.63]	10.2 [4.21–22.37]	0.406
Presepsin (pg/mL)	483 [277–1130]	1380 [924–5219]	399 [254–707]	< 0.001
Procalcitonin (ng/mL)	0.87 [0.20–17.1]	25.06 [0.42–103.73]	0.66 [0.14–2.06]	0.056
Aspartate transaminase (U/L)	24.5 [19.8–35.3]	44 [24–133]	24 [18–29]	0.003
Alanine transaminase (U/L)	17 [11.8–30.0]	19 [15–52]	17 [11–24]	0.049
γ-glutamyl transpeptidase (U/L)	31 [21.0–53.3]	70 [43–74]	26 [20–43]	0.002
Creatinine (mg/dL)	1.24 [0.87–1.99]	1.85 [1.19–3.86]	1.05 [0.85–1.84]	0.020
sex (male/female)	33/17	8/3	25/14	0.440
placement of urinary catheter (Y/N)	24/26	6/5	18/21	0.623
urological cancer (Y/N)	15/35	1/10	14/25	0.085
urinary calculi (Y/N)	12/38	4/7	8/31	0.240
DM (Y/N)	9/41	3/8	6/33	0.308
internal use of steroid (Y/N)	9/41	3/8	6/33	0.308

Values are expressed as number or median [interquartile range, IQR]

*DM* diabetes mellitus, *Y* yes, *N* No

### Prediction of SIRS on the enrollment day

The overall median baseline presepsin, PCT, and CRP levels were 483 pg/mL, 0.87 ng/mL, and 10.45 mg/L, respectively, with no significant difference between the SIRS and non-SIRS groups (Additional file 1: Table S1).”

### Prediction of septic shock on the enrollment day

**Fig. 1:**
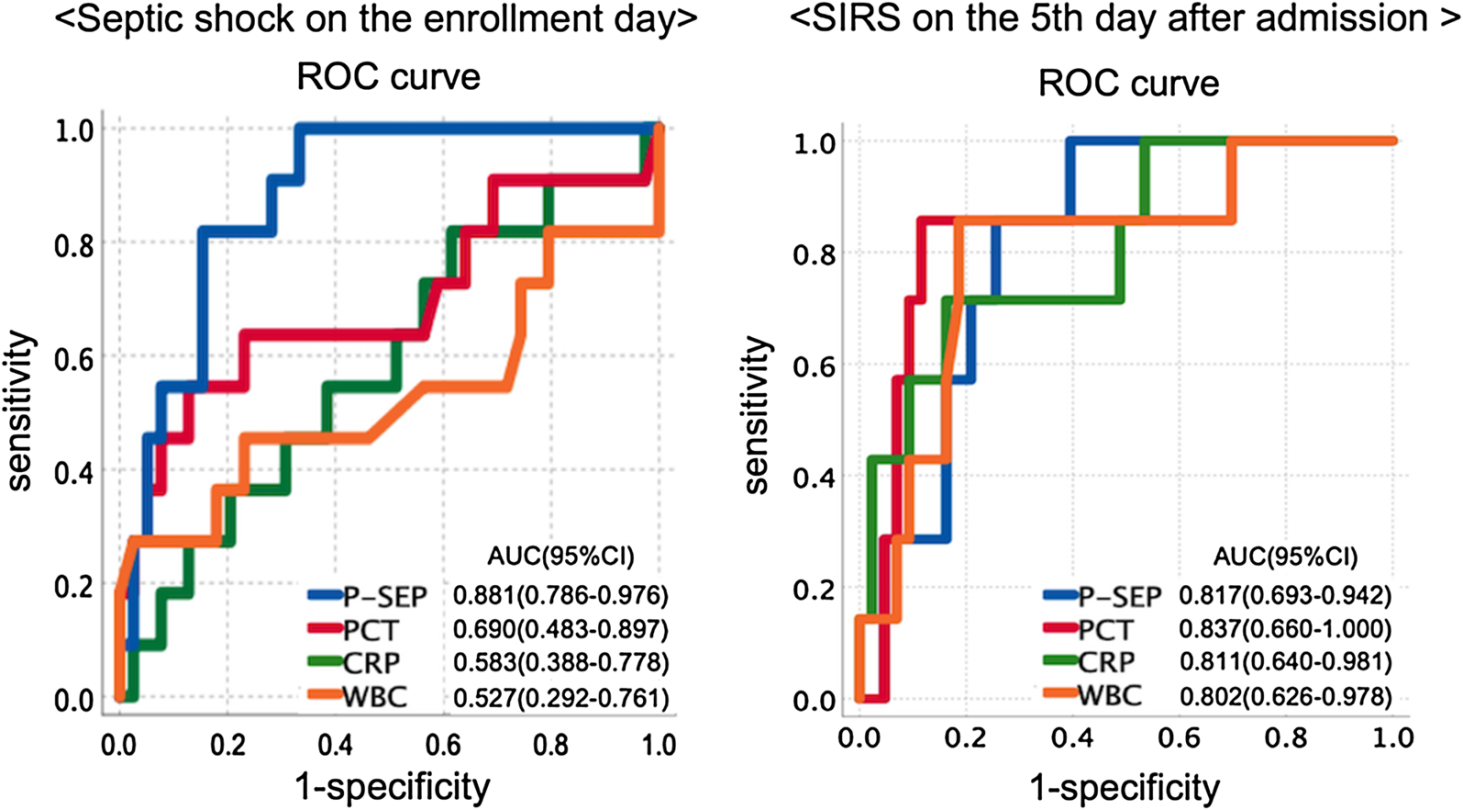
ROC curve for presepsin, procalcitonin, CRP, and WBC in patients with definitive diagnosis of septic shock on the enrollment day and SIRS on the 5th day after admission. Abbreviations: P-SEP; presepsin, PCT; procalcitonin, CRP; C-reactive protein, WBC; white blood cell

The median presepsin level (pg/mL) was significantly higher in the septic shock group (1380 vs. 399, *p* < 0.001). The PCT and CRP levels were not significantly different between the septic shock and non-septic shock groups. Other blood test results that were significantly higher in the septic group included: AST (*p* = 0.003), ALT (*p* = 0.049), γGTP (*p* = 0.002 and Cr (*p* = 0.02), respectively (Table 1). Logistic regression analysis for evaluating factors associated with septic shock on the enrollment day is shown in Table 2. The factors associated with septic shock on univariate analysis included presepsin (≥ 500 pg/mL), PCT (≥ 2 ng/ml), AST (≥ 34 U/L) and γGTP (≥ 47 U/L). Only presepsin level retained a significant value after adjusting for confounders using multivariate logistic regression analysis (Table 2). ROC curve for diagnosing septic shock indicated an AUC of 0.881 for presepsin, which was greater than these for other markers (Fig. 1). The Cutoff level of presepsin with the optimum diagnostic efficiency by the ROC curves was 492 ng/ml, which was broadly similar to the clinical cut off value (500 pg/mL).

**Table 2:** Prediction of septic shock on the enrollment day by logistic regression analysis

Variable	Univariate analysis			Multivariable analysis		
	*P *value	HR	95% CI	*P* value	HR	95% CI
Sex (male vs female)	0.595					
WBC (4000–12,000 vs other)	0.497					
CRP (≥ 0.5 vs < 0.5)	0.360					
CRP (≥ 10 vs < 10)	0.848					
Presepsin (≥ 500 vs < 500)	0.007	20.000	2.305–173.553	0.029	12.157	1.298–113.892
PCT (≥ 0.05 vs < 0.05)	0.630					
PCT (≥ 2 vs < 2)	0.025	5.075	1.223–21.065	0.673		
AST (≥ 34 vs < 34)	0.006	8.000	1.829–34.996	0.375		
ALT (≥ 28 vs < 28)	0.105					
γGPT (≥ 47 vs < 47)	0.005	8.889	1.941–40.711	0.081		
Cr (male; ≥ 1.07 vs < 1.07, female; ≥ 0.79 vs < 0.79)	0.095					
placement of urinary catheter (Y vs N)	0.624					
urological cancer (Y vs N)	0.118					
urinary calculi (Y vs N)	0.284					
DM (Y vs N)	0.371					
internal use of steroid (Y vs N)	0.371					

*WBC* white blood cell, *CRP* C-reactive protein, *PCT* procalcitonin, *AST* aspartate transaminase, *ALT* Alanine transaminase, *γGTP* γ-glutamyl transpeptidase, *Cr* creatinine, *DM* diabetes mellitus, *Y* yes, *N* no

### Prediction of SIRS on the 5th day after admission

The median presepsin level (pg/mL) was significantly higher in the SIRS group on the 5th day after admission (day1; 1167 vs. 419, *p* < 0.001, day3; 633 vs. 311, *p* = 0.027). The PCT (day1: 73.1 vs. 0.55, *p* = 0.003; day3: 20.34 vs. 1.35, *p* = 0.005)) and CRP (day1: 25.63 vs. 8.23, *p* = 0.007; day3: 25.58 vs. 10.71, *p* < 0.001) levels were also significantly different between the SIRS and non-SIRS groups on the 5th day after admission. Other blood test results that were significantly higher in the SIRS group on the 5th day after admission included: day1/AST (34 vs. 24, *P* = 0.013); day1/Cr (2.3 vs. 1.05, *p* = 0.007); day1/WBC (18,200 vs. 13,600, *p* = 0.009); and day3/WBC (11,900 vs. 8400, *p* = 0.021) (Table 3). The ratio of positive blood culture (86% vs. 42%, *p* = 0.039) and urinary calculi (57% vs. 19%, *p* = 0.048) significantly differed between the SIRS and non-SIRS groups on the 5th day after admission. Logistic regression analysis to evaluate factors associated with SIRS on the 5th day after admission is shown in Table 4. These factors included day1/presepsin (≥ 500 pg/mL), day1/PCT (≥ 2 ng/ml) and urinary calculi. Only day1/PCT level retained statistical significance after adjusting for confounders using multivariate logistic regression analysis (Table 4). ROC curve in patients with definitive diagnosis of SIRS on the 5th day after admission indicated an AUC of 0.837 for PCT, which is larger than these for other markers (Fig. 1). Regarding presepsin, the cutoff level with the optimum diagnostic efficiency using the ROC curves was 492 ng/ml, which was broadly similar to the clinical cut off value (500 pg/mL).

**Table 3:** Characteristics of Patients with or without SIRS on the 5th day

Variable	SIRS group	Non-SIRS group	*p*-value
	N = 7	N = 43	
Age (years)	64 [54–68]	68 [61–77]	0.126
Day1/White blood cell (/mm^3^)	18,200 [17,200–24900]	13,600 [9900–16,000]	0.009
Day1/C-reactive protein (mg/L)	25.63 [9.58–31.38]	8.23 [4.1–16.6]	0.007
Day1/Presepsin (pg/mL)	1167 [878–5129]	419 [277–922]	0.006
Day1/Procalcitonin (ng/mL)	73.1 [25.06–103.73]	0.55 [0.14–2.06]	0.003
Day1/Aspartate transaminase (U/L)	34 [28–126]	24 [18–33]	0.013
Day1/Alanine transaminase (U/L)	23 [18–52]	16 [11–25]	0.056
Day1/γ-glutamyl transpeptidase (U/L)	48 [38–72]	28 [21–51]	0.133
Day1/Creatinine (mg/dL)	2.30 [1.72–2.71]	1.05 [0.85–1.84]	0.007
Day3/White blood cell (/mm^3^)	11,900 [9400–20,100]	8400 [5900–10,400]	0.021
Day3/C-reactive protein (mg/L)	25.58 [16.47–34.54]	10.71 [6.55–15.40]	< 0.001
Day3/Presepsin (pg/mL)	633 [464–1360]	311 [196–695]	0.027
Day3/Procalcitonin (ng/mL)	20.34 [14.23–32.27]	1.35 [0.30–15.09]	0.005
Sex (male/female)	4/3	29/14	0.446
Urinary culture (P/N)	7/0	38/5	0.454
Blood culture (P/N)	6/1	18/25	0.039
Placement of urinary catheter (Y/N)	2/5	22/21	0.244
Urological cancer (Y/N)	1/6	14/29	0.311
Urinary calculi (Y/N)	4/3	8/35	0.048
DM (Y/N)	1/6	8/35	0.630
Surgical procedure (Y/N)	6/1	22/21	0.095
Internal use of steroid (Y/N)	2/5	7/36	0.370

Values are expressed as number or median [interquartile range, IQR]

*DM* diabetes mellitus, *P* positive, *N* no, *Y* yes

**Table 4:** Prediction of SIRS on the 5th day by logistic regression analysis

Variable	Univariate analysis			Multivariable analysis		
	*P*-value	HR	95% CI	*P*-value	HR	95% CI
Day1/WBC (4000–12,000 or other)	0.19					
Day1/CRP (≥ 10 or < 10)	0.280					
Day1/Presepsin (≥ 500 or < 500)	0.049	9.176	1.013–83.108	0.366		
Day1/PCT (≥ 2 or < 2)	0.012	17.455	1.886–161.528	0.012	17.455	1.886–161.528
Day1/AST (≥ 34 or < 34)	0.079					
Day1/ALT (≥ 28 or < 28)	0.283					
Day1/γGPT (≥ 47 or < 47)	0.177					
Day1/Cr (male; ≥ 1.07 or < 1.07, female; ≥ 0.79 or < 0.79)	0.998					
Day3/WBC (4000–12,000 or other)	0.120					
Day3/CRP (≥ 10 or < 10)	0.998					
Day3/Presepsin (≥ 500 or < 500)	0.067					
Day3/PCT (≥ 2 or < 2)	0.998					
Sex (male or female)	0.596					
Urinary culture (P vs N)	0.999					
Blood culture (P vs N)	0.059					
Placement of urinary catheter (Y vs N)	0.280					
Urological cancer (Y vs N)	0.346					
Urinary calculi (Y vs N)	0.040	5.833	1.084–31.377	0.182		
DM (Y vs N)	0.783					
Surgical procedure (Y vs N)	0.120					
Internal use of steroid (Y vs N)	0.439					

*WBC* white blood cell, *CRP* C-reactive protein, *PCT* procalcitonin, *AST* aspartate transaminase, *ALT* alanine transaminase, *γGTP* γ-glutamyl transpeptidase, *Cr* creatinine, *DM* diabetes mellitus, *P* positive, *N* no, *Y* yes

## Discussion

In this study, elevated presepsin level on admission was an independent risk factor for septic shock, while elevated PCT on admission was independently associated with SIRS on the 5th day after admission in patients with UTI. Presepsin and PCT originate from different sources [3–5]. These results support our hypothesis that presepsin could be a useful marker for detecting severe sepsis in patients with UTI. On the other hand, PCT could predict therapeutic courses in patients with UTI better than did presepsin.

Presepsin is one of the biomarkers that increase after bacterial infections [11]. Its levels are increased in acute pyelonephritis patients with bacteremia [12]. Moreover, elevation of presepsin levels before initiating treatment might predict the development of sepsis in patients with obstructive acute pyelonephritis [13]. In this study, we evaluated the patients who needed hospitalization due to not only pyelonephritis, but also prostatitis. Additionally, elevation of presepsin levels on the enrollment day was a predictor of septic shock. Therefore, presepsin might be a useful for detecting severe urosepsis that needs vasopressor therapy.

Sever sepsis and septic shock can be fatal, with mortality rates: 28.3% in the United States and 41.1% in Europe [2]. Clinically, it is very useful to predict septic shock on admission. Therefore, it is essential that biomarkers increase immediately after infections and have high sensitivity for sepsis to be reliable. Presepsin levels have been shown to increase within 2 h together with blood bacterial counts, and peak at 3 h after infection [6]. On the other hand, elevated PCT and CRP levels are detected within 3–6 and 6 h, and peak at 6–8 and 36–50 h, respectively [14, 15]. It has also been reported that patients with severe sepsis had significantly higher presepsin levels than did those with sepsis, local infection, or SIRS [16]. In this study, elevation of presepsin levels on the enrollment day was a predictor of septic shock, not of SIRS. These results suggest that presepsin may be used to identify patients at increased risk of more severe infections at early stages.

One important characteristic of biomarkers is their prognostic values. Presepsin levels on day 1 were reported to be correlated with 60-day in-hospital mortality in patients with sepsis, severe sepsis, or septic shock [17]; a longer intensive-care unit stay; and a lower degree of resolution of primary infection [8, 9]. Mortality is one of the prognostic variables. Since there were no mortalities in this current study, we used presence or absence of SIRS on day 5 for prognosis. In this study, the levels of presepsin were significantly higher in the SIRS group than in the non-SIRS group on day 5 after admission. However, the levels of PCT on day1 were only identified as a predictor of SIRS on day5 using multivariate logistic regression analysis. Regarding PCT, the AUC for PCT to predict 30-day mortality in patients with febrile UTI was reported to be 0.71 (95% CI: 0.56–0.85) [18]. There are a few reports that compared between presepsin and PCT for evaluating their prognostic values in patients with UTI. Further research that includes a large sample size of patients with severe urosepsis than does this study is needed to evaluate the reliability of each of these biomarkers for predicting treatment outcomes.

This study has several limitations. First, since 2016, sepsis has been defined according to the Sequential (Sepsis-Related) Organ Failure Assessment (SOFA) [19]. This study began in 2014, and we did not assess the level of consciousness, which is a requirement for the SOFA scoring. Therefore, instead of the SOFA, SIRS criteria [10] were used for defining sepsis and septic shock. Second, this study might have been limited by its single-center design and small sample size. Third, we did not consider renal function in setting the reference values of presepsin and PCT. The levels of presepsin and PCT have been previously reported to be affected by renal function [20, 21]. It would have been ideal to set the reference values depending on assessment of renal function. However, these adjustments are yet to be clarified. Fourth, all patients were discharged from our hospital alive in this study. However, it has been reported that severe sepsis and septic shock could be fatal, with mortality rate of 28.3%-41.1% [2]. Regarding 11 patients with septic shock on the enrollment day, seven patients were without SIRS on the fifth day. Six patients underwent surgical procedures out of these seven patients, and it seems that the combination of antibiotics and surgical procedures might have been quite effective. There is a possibility that these patients might have not needed any vasopressors infusion in the course of treatments. Moreover, the number of patients was not enough to evaluate presepsin for detecting severe sepsis and predicting the therapeutic course in comparison with PCT and CRP. Further research with a larger number of patients is needed.

## Conclusions

This study showed that, in patients with UTI, presepsin may be a good marker for diagnosis of septic shock based on the data of admission.

## Supplementary Information

**Additional file 1. Supplemental Table 1.** Characteristics of Patients with or without SIRS on the enrollment day.

## Data Availability

The datasets used and/or analysed during the current study are available from the corresponding author on reasonable request.

## Abbreviations

ALT: Aspartate transaminase
AST: Alanine transaminase
AUC: The area under the curve
Cr: Creatinine
CRP: C-reactive protein
γGPT: γ-Glutamyl transpeptidase
PCT: Procalcitonin
ROC: A receiver operating characteristics
SIRS: Systemic inflammatory response syndrome
SOFA: The Sequential Organ Failure Assessment
UTI: Urinary tract infections
WBC: White blood cell
