# Supplementary material for: Presepsin as a predictor of septic shock in patients with urinary tract infection
Source: BMC Urol. 2021 Oct 12;21:144. doi: 10.1186/s12894-021-00906-4 (PMC8513358; doi:10.1186/s12894-021-00906-4)
Supplement: Supplementary file 1 — Additional file 1. Supplemental Table 1. Characteristics of Patients with or without SIRS on the enrollment day. [file 12894_2021_906_MOESM1_ESM.docx]

| Supplemental Table 1.  Characteristics of Patients with or without SIRS on the enrollment day | | | |
| --- | --- | --- | --- |
|  | SIRS group | Non-SIRS group |  |
| Variable | N=39 | N=11 | p-value |
| Age (years) | 66 [57-73] | 77 [62-81] | 0.049 |
| C-reactive protein (mg/L) | 10.7 [4.2-24.2] | 10.2 [4.1-14.6] | 0.708 |
| Presepsin (pg/mL) | 541 [285-1161] | 357 [221-707] | 0.276 |
| Procalcitonin (ng/mL) | 0.66 [0.16-25.0] | 1.04 [0.51-1.73] | 0.770 |
| Aspartate transaminase (U/L) | 24 [20-36] | 25 [18-33] | 0.664 |
| Alanine transaminase (U/L) | 17 [11-30] | 15 [12-25] | 0.432 |
| γ-glutamyl transpeptidase (U/L) | 32 [21-54] | 30 [21-42] | 0.860 |
| Creatinine (mg/dL) | 1.19 [0.92-2.17] | 1.44 [0.84-1.74] | 0.419 |
| sex (male/female) | 27/12 | 6/5 | 0.287 |
| placement of urinary catheter (Y/N) | 19/20 | 5/6 | 0.848 |
| urological cancer (Y/N) | 11/28 | 4/7 | 0.430 |
| urinary calculi (Y/N) | 10/29 | 2/9 | 0.472 |
| DM (Y/N) | 8/31 | 1/10 | 0.353 |
| internal use of steroid (Y/N) | 7/32 | 2/9 | 0.645 |

Values are expressed as number or median [interquartile range, IQR].

DM; diabetes mellitus, Y; yes, N; No,
